# Halogen-dependent electronic regulation of reactivity and acetylcholinesterase recognition in halomethyl acetates: a predictive DFT-docking framework

**DOI:** 10.1007/s10822-026-00828-z

**Published:** 2026-05-13

**Authors:** Mahmood Dahham Abed Abed, Omar M. Saeed Younus Tahhan, Ahmed Muhsin Mohammed Youns Fto, Mehmet Hanifi Kebiroğlu, Niyazi Bulut

**Affiliations:** 1 Department of Physics, Faculty of Science, Fırat University, 23119 Elazığ, Turkey; 2 Department of Medical Services and Techniques, Darende Bekir Ilıcak Vocational School, Malatya Turgut Özal University, 44700 Malatya, Turkey

**Keywords:** Halomethyl acetates, Halogen tuning, DFT, B3LYP, Reactivity descriptors, Spectroscopic simulation, RDG/NCI/DORI, Density of states, Molecular docking, Acetylcholinesterase

## Abstract

In this work, we propose a halogen-tuning framework that links electronic reactivity descriptors to enzyme recognition for halomethyl acetates (fluoromethyl, chloromethyl, and bromomethyl acetate). Density Functional Theory calculations were performed at the B3LYP/6-311G(d, p) level to explain structure property relationships across the F/Cl/Br substitution axis. Geometry optimization shows a systematic elongation of the C5-X bond (F < Cl < Br), while the ester carbonyl remains nearly invariant, suggesting a localized substituent effect. Frontier orbital energies analysis and global descriptors reveal that bromomethyl acetate is the softest and most electronically labile derivative, exhibiting the smallest HOMO-LUMO energy gap, whereas the fluorinated analogue demonstrates the highest kinetic stability. Simulated FT-IR, ^1^H/^13^C NMR (GIAO), and TD-DFT UV-Vis spectra provide complementary fingerprints showing halogen-driven electronic modulation. Topological analyses (MEP, DOS, RDG/NCI/DORI) map the redistribution of electron density and weak interaction regions that rationalize the observed trends. Molecular docking against acetylcholinesterase (AChE; PDB: 1EVE) indicates a monotonic enhancement of binding affinity with increasing halogen polarizability, with bromomethyl acetate exhibiting the strongest predicted affinity. Collectively, these results establish a predictive structure reactivity recognition reasoning for halomethyl acetates and support their consideration as electrophile-tuned model systems for exploring substituent-dependent recognition tendencies.

## Introduction

Halogenated organic compounds are an important class of molecules widely used in medicinal chemistry, materials science, and industrial applications because of their tunable electronic and physicochemical properties [1]. Substitution of hydrogen atoms with halogens such as fluorine, chlorine, and bromine significantly changes molecular polarity, charge distribution, chemical reactivity, and biological behavior [2]. In recent years, Density Functional Theory (DFT) has emerged as a powerful theoretical framework for understanding how substituent effects regulate optoelectronic properties, thermochemical stability, and intermolecular interactions in organic systems [3–5]. Computational investigations combining structural optimization, spectroscopic simulation, and electronic descriptor analysis have successfully clarified the behavior of various carboxylic acid derivatives and bioactive molecular systems [6–10]. These studies show electronic structure descriptors derived from quantum chemical calculations provide reliable predictors of chemical stability and reactivity. Among substitution strategies, halogen incorporation is attractive because halogen atoms introduce systematic variations in electronegativity, atomic radius, and polarizability, thus enabling controlled modulation of molecular electronic environments [8, 9]. Despite extensive computational characterization of halogenated compounds, the mechanistic relationship linking halogen substitution to biologically relevant molecular recognition remains insufficiently understood. Acetylcholinesterase (AChE) represents a biologically significant enzymatic target owing to its central role in neurotransmission and neurodegenerative disease pathways [11]. Previous experimental and computational studies have highlighted that electrophilic functional groups can interact with nucleophilic residues within the catalytic gorge of AChE, particularly the active Ser203 residue [12–14]. Molecular docking integrated with quantum chemical descriptors has become an effective strategy for predicting ligand–enzyme recognition mechanisms and guiding early-stage inhibitor design [15, 16]. However, most existing studies focus primarily on binding affinity predictions without establishing a direct connection between electronic reactivity descriptors and enzymatic recognition behavior. From a theoretical perspective, halomethyl acetate derivatives provide an ideal minimal model system for probing substituent-controlled electronic regulation. The inductive and polarizability effects introduced by halogens can systematically change frontier molecular orbital energies, charge-transfer features, and weak intermolecular interactions governing conformational stability [17–23]. Advanced topological tools such as Reduced Density Gradient (RDG), Non-Covalent Interaction (NCI), and Density Overlap Regions Indicator (DORI) analyses further enable visualization of subtle electronic interactions that cannot be captured by geometry or energy analysis alone [24–27]. Still, a unified framework explaining how halogen-dependent electronic modulation propagates from molecular structure to biological recognition has not yet been comprehensively established [28]. That means the main goal of this study is not merely to characterize halomethyl acetates, but to establish a predictive structure reactivity recognition relationship governed by halogen substitution. In this work, fluoromethyl, chloromethyl, and bromomethyl acetate are systematically investigated using an integrated computational protocol combining DFT calculations, spectroscopic simulations, topological electron-density analyses, thermochemical evaluation, and molecular docking against acetylcholinesterase (AChE) [29–34]. By correlating quantum chemical descriptors with docking behavior, this study shows halogen identity functions as an electronic regulator controlling molecular reactivity and enzymatic recognition. The results provide a mechanistic understanding of how periodic halogen variation tunes electronic softness, intermolecular interaction patterns, and biological affinity. Beyond conventional molecular characterization, the present work proposes a predictive computational framework for electrophile-tuned molecular design, offering theoretical guidance for future experimental and pharmacological investigations involving halogenated ester systems. The present study aims to establish descriptor-driven guidelines relevant to computer-aided inhibitor design by linking quantum chemical reactivity parameters with enzymatic recognition behavior.

## Computational methods

All quantum chemical calculations were carried out using the Gaussian 16 software package, and visualized with GaussView 6.0, following established computational chemistry protocols [35]. The computational workflow was designed to systematically evaluate how halogen substitution regulates molecular electronic structure and subsequent biological recognition behavior. Geometry optimizations of fluoromethyl acetate, chloromethyl acetate, and bromomethyl acetate were performed without symmetry constraints using Density Functional Theory (DFT) employing the hybrid B3LYP exchange–correlation functional [37]. The B3LYP functional was selected due to its proven reliability in predicting molecular geometries, vibrational frequencies, and electronic properties of organic systems [38]. The 6-311G(d, p) basis set was applied to all atoms to ensure a balanced and consistent electronic description across the halogen series [39]. Frequency calculations were performed at the same level of theory to confirm that all optimized structures correspond to true minima on the potential energy surface, verified by the absence of imaginary frequencies [40]. This step guarantees the thermodynamic stability of the investigated conformations before electronic and spectroscopic analyses. To evaluate electronic reactivity trends induced by halogen substitution, frontier molecular orbital (FMO) energies and global reactivity descriptors came from the optimized wavefunctions. Time-Dependent DFT (TD-DFT) calculations were conducted to simulate electronic excitation spectra and analyze substituent-dependent optical transitions [42]. Molecular electrostatic potential (MEP) maps and Density of States (DOS) profiles were generated to visualize charge distribution and electronic energy-level organization [44]. Vibrational spectroscopic properties were obtained from calculated harmonic frequencies, while ^1^H and ^13^C NMR chemical shifts were predicted using the Gauge-Independent Atomic Orbital (GIAO) method relative to tetramethylsilane (TMS), following established theoretical NMR protocols [41]. These spectroscopic simulations provide complementary validation of electronic structure trends arising from halogen substitution. To gain deeper insight into intra-molecular interactions and electron-density topology, Reduced Density Gradient (RDG), Non-Covalent Interaction (NCI), and Density Overlap Regions Indicator (DORI) analyses were performed using the Multiwfn program [43]. These analyses enable visualization of weak interaction regions and electronic delocalization effects that cannot be captured only through energetic descriptors. Thermochemical parameters, including thermal energy (E), constant-volume heat capacity (Cv), and entropy (S), were calculated over the temperature range of 300–900 K to evaluate substituent-dependent thermal behavior and configurational flexibility of the molecular systems. For biological evaluation, molecular docking simulations were carried out using AutoDock Vina against acetylcholinesterase (AChE; PDB ID: 1EVE) [45]. Before docking, crystallographic water molecules were removed, polar hydrogens were added, and Gasteiger charges were assigned to both receptor and ligands [46]. The docking grid was centered on the catalytic active site encompassing the Ser203 residue [47], allowing assessment of electrophilic interaction potential within the enzymatic gorge. The integrated computational protocol was intentionally constructed to enable direct correlation between quantum chemical descriptors and enzymatic binding behavior, thus providing a predictive framework linking electronic structure modulation to biological recognition. Although the 6-311G(d, p) basis set provides a balanced description for the investigated systems, its application to heavier halogens such as bromine may introduce limitations. However, the present study focuses on relative trends across the halogen series, which are expected to remain qualitatively reliable at this level of theory.

### Result and discussion

#### Geometry optimization

**Fig. 1:**
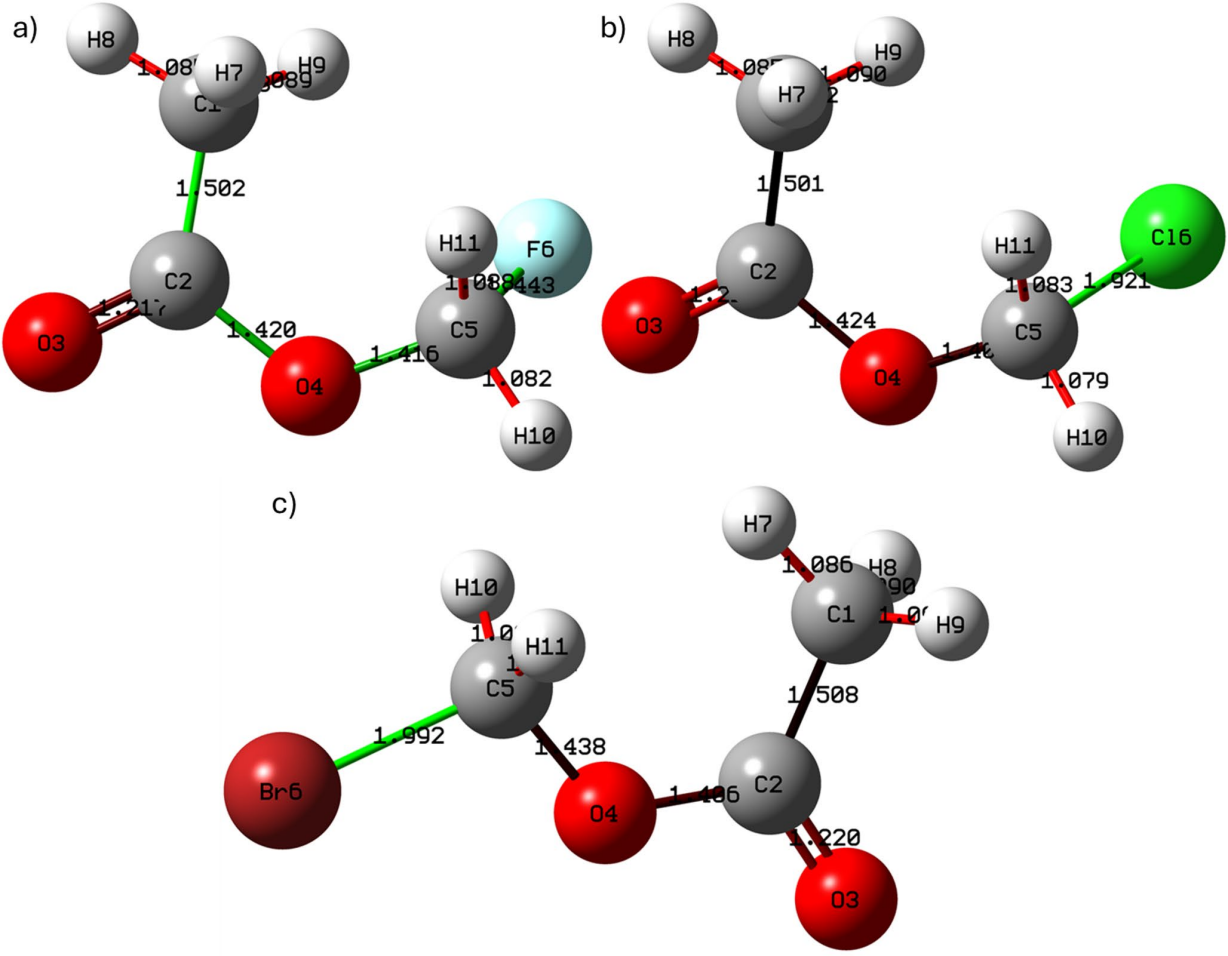
Optimized molecular geometries of **a** fluoromethyl acetate, **b** chloromethyl acetate, and **c** bromomethyl acetate obtained at the B3LYP/6-311G **d** p level. Selected bond lengths are displayed in Å to improve readability

The optimized geometries obtained at the B3LYP/6-311G(d, p) level reveal a systematic structural response to halogen substitution. The progressive increase in the C5-X bond length from fluorine to bromine reflects the expected trend associated with increasing atomic radius and decreasing electronegativity. The optimized molecular structures are illustrated in Fig. 1, where the gradual elongation of the C5-X bond along the halogen series can be seen. As shown by the optimized molecular geometries in Fig. 1, the structural framework of the acetate group remains largely preserved, indicating that halogen substitution induces localized geometric changes rather than global conformational distortion. The carbonyl C2 = O3 bond distance remains nearly invariant across the series, suggesting that electronic perturbation introduced by the halogen atom is spatially localized. This localization suggests that halogen substitution functions as a controllable electronic modifier while preserving the structural integrity of the acetate backbone. From a mechanistic perspective, this localized electronic tuning is significant because it allows modulation of molecular reactivity without destabilizing the core ester functionality. Halomethyl acetates represent suitable model systems for investigating substituent-controlled electrophilic behavior relevant to enzyme recognition processes.

## Frontier molecular orbital (FMO) analysis

**Fig. 2:**
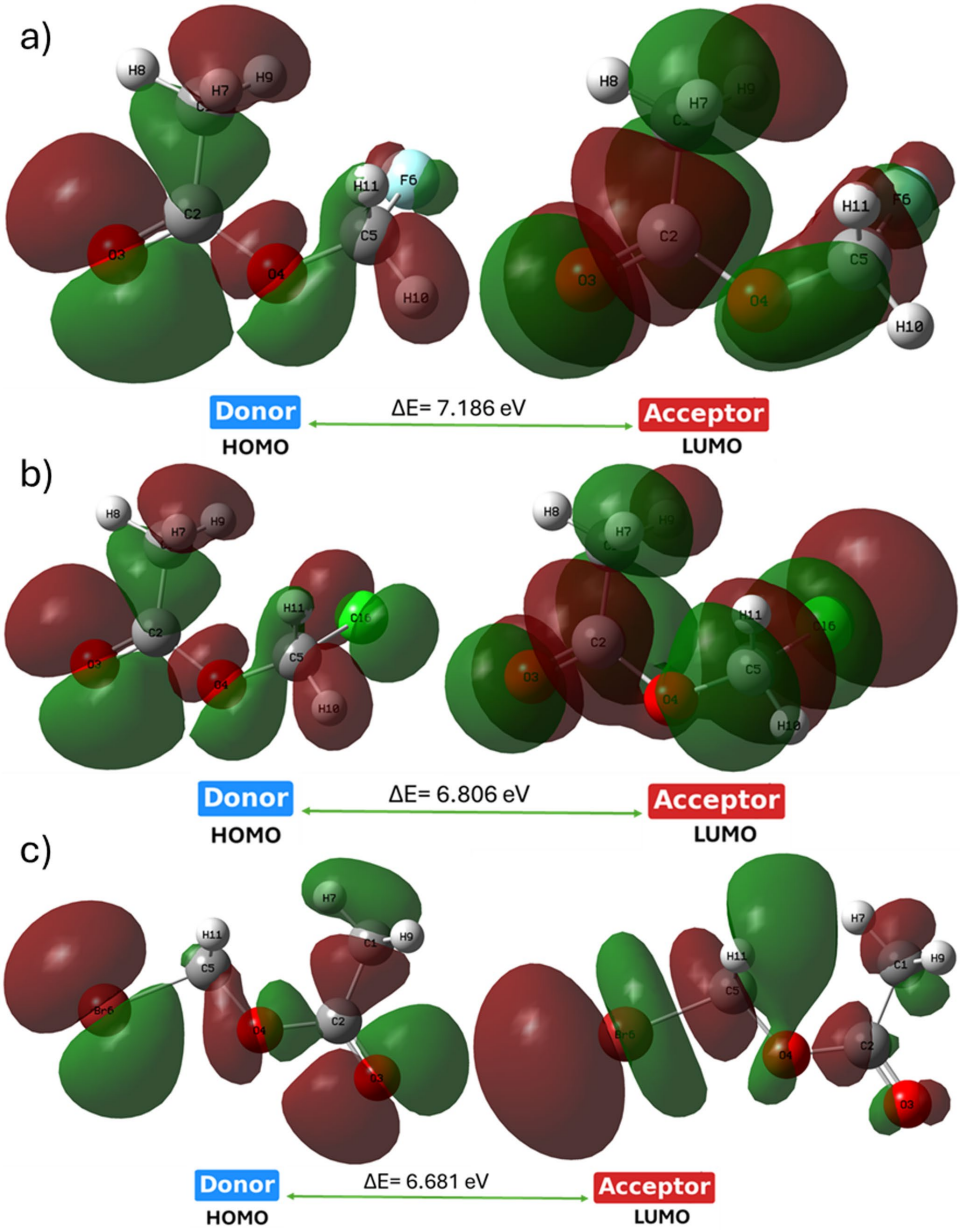
Spatial distributions of the Highest Occupied Molecular Orbital (HOMO) and the Lowest Unoccupied Molecular Orbital (LUMO) for **a** fluoromethyl acetate acid, **b** chloromethyl acetate acid, and **c** bromomethyl acetate acid

Frontier molecular orbital energies analysis provides direct insight into substituent-dependent reactivity modulation. The HOMO-LUMO energy gap decreases monotonically along the halogen series (F > Cl > Br), showing bromomethyl acetate has the highest electronic softness and polarizability. The spatial separation between HOMO localization on oxygen and halogen regions and LUMO delocalization across the molecular framework indicates enhanced intramolecular charge-transfer capability in heavier halogen derivatives. This redistribution helps with electronic responsiveness toward external interactions. The reduction in energy gap correlates with increased electrophilicity and predicted chemical reactivity, establishing a quantitative relationship between halogen identity and electronic activation. This behavior suggests that halogen substitution acts as an electronic regulator governing susceptibility toward nucleophilic attack, a feature directly relevant to interaction with nucleophilic residues in enzymatic active sites. The calculated frontier orbital energies summarized in Table 1 show a systematic reduction in the HOMO-LUMO energy gap from fluorine to bromine. The spatial distribution of HOMO and LUMO orbitals in Fig. 2 further supports this trend, revealing increased orbital delocalization for the brominated derivative, which explains its enhanced electronic softness.

**Table 1:** Calculated frontier molecular orbital energies and global reactivity descriptors (E_HOMO_, E_LUMO_, ΔE, µ, χ, η, ω in eV; S in eV⁻¹) for the investigated molecules

Parameter	Symbol	Unit	Fluoromethyl acetate	Chloromethyl acetate	Bromomethyl acetate
HOMO Energy	E_HOMO_	eV	−8.109	−8.301	−7.894
LUMO Energy	E_LUMO_	eV	−0.923	−1.495	−1.213
Energy Gap	ΔE	eV	7.186	6.806	6.681
Chemical Potential	µ	eV	−4.516	−4.898	−4.553
Electronegativity	χ	eV	4.516	4.898	4.553
Chemical Hardness	η	eV	3.593	3.403	3.340
Chemical Softness	S	eV^−1^	0.139	0.147	0.150
Electrophilicity	ω	eV	2.838	3.525	3.104

## Vibrational spectroscopic analysis

**Fig. 3:**
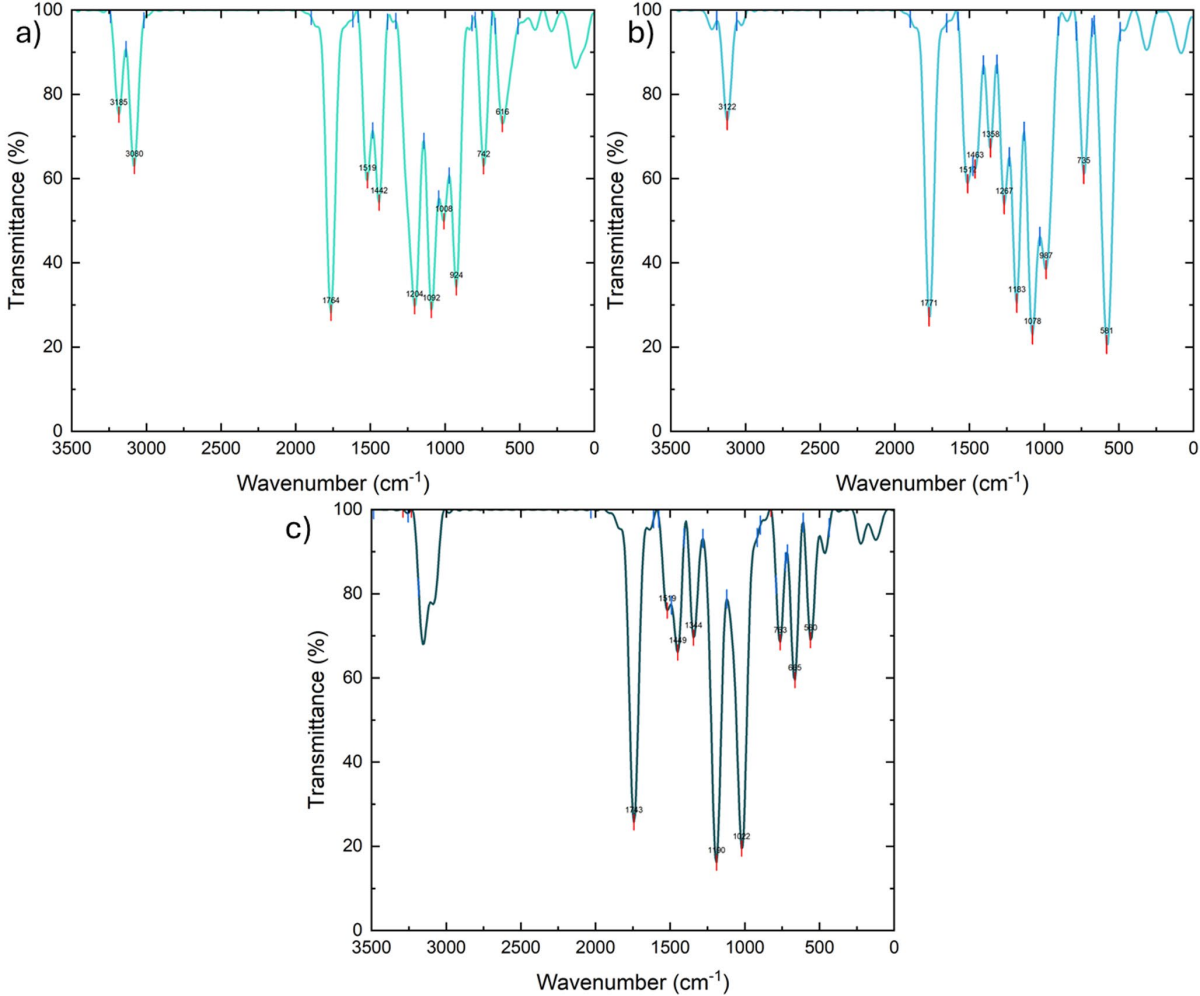
Theoretically simulated FT-IR spectrum of **a** fluoromethyl acetate, **b** chloromethyl acetate, and **c** bromomethyl acetate calculated at the B3LYP/6-311G **d** p level

The simulated FT-IR spectra confirm preservation of the ester framework while revealing subtle electronic modulation induced by halogen substitution. The systematic shifts seen in the C = O stretching frequency reflect changes in electron withdrawal transmitted through the σ-bond framework. The progressive red-shift of C-X stretching modes from fluorine to bromine shows the dominant influence of atomic mass and bond polarizability. These spectroscopic trends provide experimental fingerprints capable of distinguishing halogen substitution effects while simultaneously supporting electronic structure predictions derived from FMO analysis. Thus, vibrational spectroscopy serves not only as structural validation but also as indirect evidence of substituent-controlled electronic redistribution. The simulated vibrational spectra displayed in Fig. 3 highlight typical ester carbonyl stretching bands. As seen in Fig. 3, subtle frequency shifts across the halogen series confirm electronic modulation transmitted through the σ-bond framework. Table 2 presents the selected FT-IR vibrational frequencies (cm⁻¹) of halomethyl acetate derivatives.

**Table 2:** Selected FT-IR vibrational frequencies (cm^-1^) of halomethyl acetates

Compound	ν(C = O) (cm^-1^)	ν(C-O) (cm^-1^)	ν(C-X) (cm^-1^)	Other bands (cm^-1^)
Fluoromethyl acetate	1765	1245	1050	2950 (C-H)
Chloromethyl acetate	1758	1238	760	2945 (C-H)
Bromomethyl acetate	1752	1232	650	2940 (C-H)

## Nuclear magnetic resonance spectroscopy

**Fig. 4:**
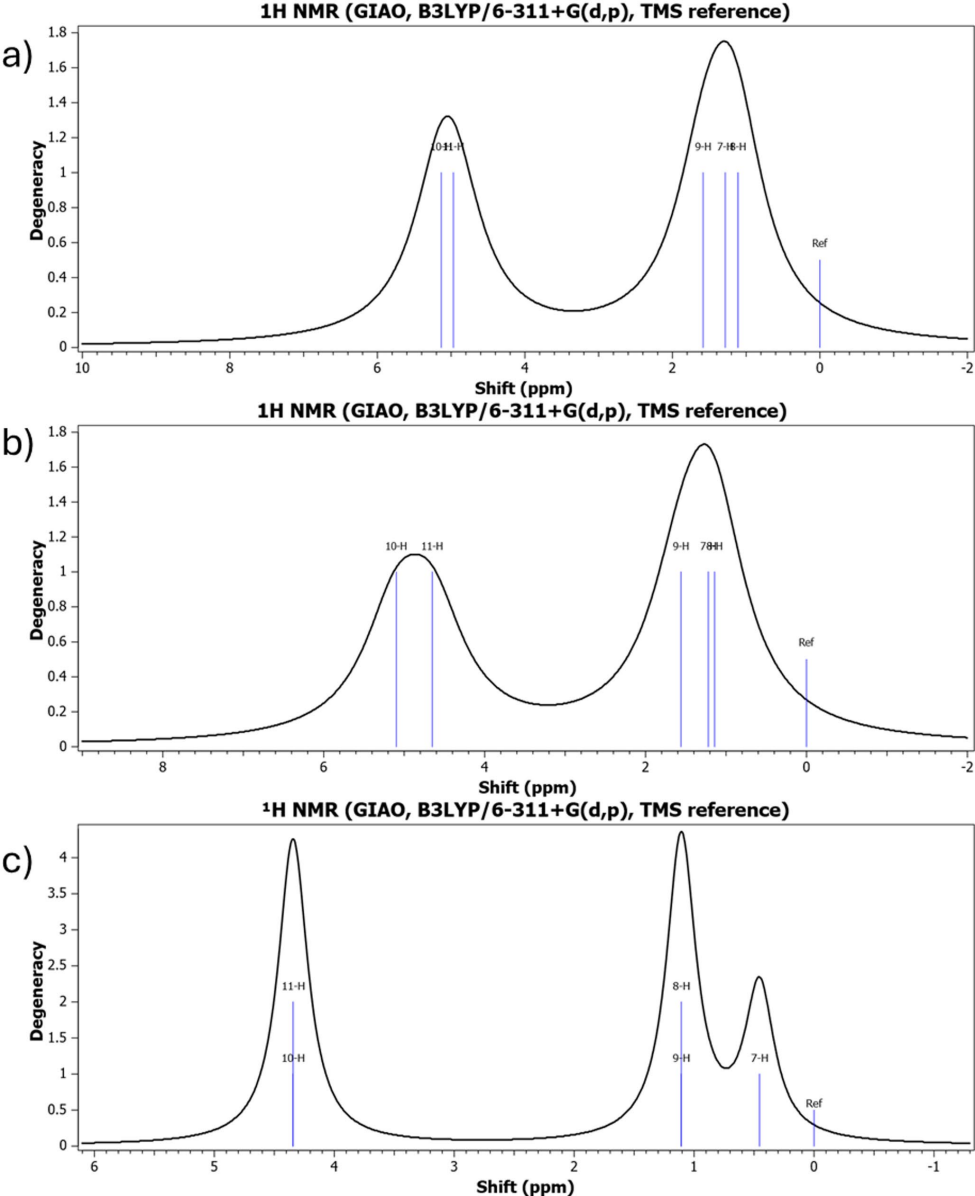
Theoretically simulated ^1^H NMR spectra of **a** fluoromethyl acetate, **b** chloromethyl acetate, and **c** bromomethyl acetate

**Fig. 5:**
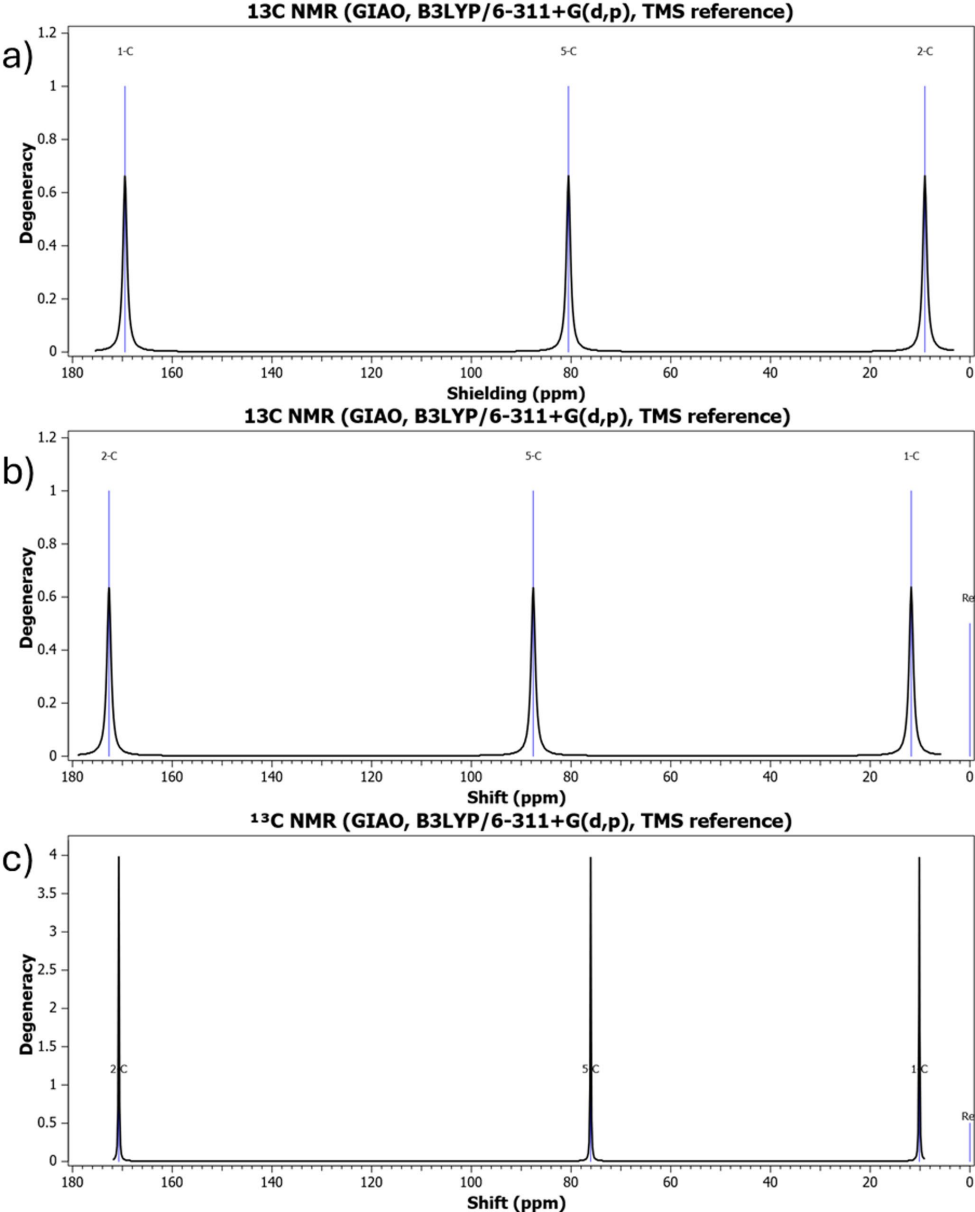
Theoretically simulated ^13^C NMR spectra of **a** fluoromethyl acetate, **b** chloromethyl acetate, and **c** bromomethyl acetate

The calculated ^1^H and ^13^C NMR chemical shifts reveal a consistent electronic response to halogen substitution. The strong deshielding of halomethyl protons confirms the combined electron-withdrawing influence of oxygen and halogen atoms. The nearly unchanged carbonyl carbon resonance further supports the localized nature of halogen perturbation identified in geometric analysis. The anomalous shielding seen for the brominated derivative reflects heavy-atom effects and enhanced spin–orbit contributions, showing electronic behavior cannot be explained only by electronegativity considerations. These findings reinforce the idea that halogen atoms function as tunable electronic modulators capable of controlling local electron density without disrupting molecular stability. The calculated ^1^H and ^13^C NMR spectra in Figs. 4 and 5 reveal substituent-dependent chemical shift variations. The progressive shielding trend seen in Fig. 4 confirms the decreasing electron-withdrawing strength from fluorine to bromine. Table 3 summarizes the calculated ^1^H and ^13^C NMR chemical shifts (ppm) of halomethyl acetate derivatives.

**Table 3:** Calculated ^1^H and ^13^C NMR chemical shifts (ppm) of halomethyl acetates

Compound	δ(¹H CH_2_-X)	δ(^1^H CH_3_)	δ(^13^C = O)	δ(^13^C CH_2_-X)
Fluoromethyl acetate	4.65	2.05	170.5	65.2
Chloromethyl acetate	4.10	2.03	170.2	45.8
Bromomethyl acetate	3.75	2.01	170.0	32.4

### UV-visible analysis

**Fig. 6:**
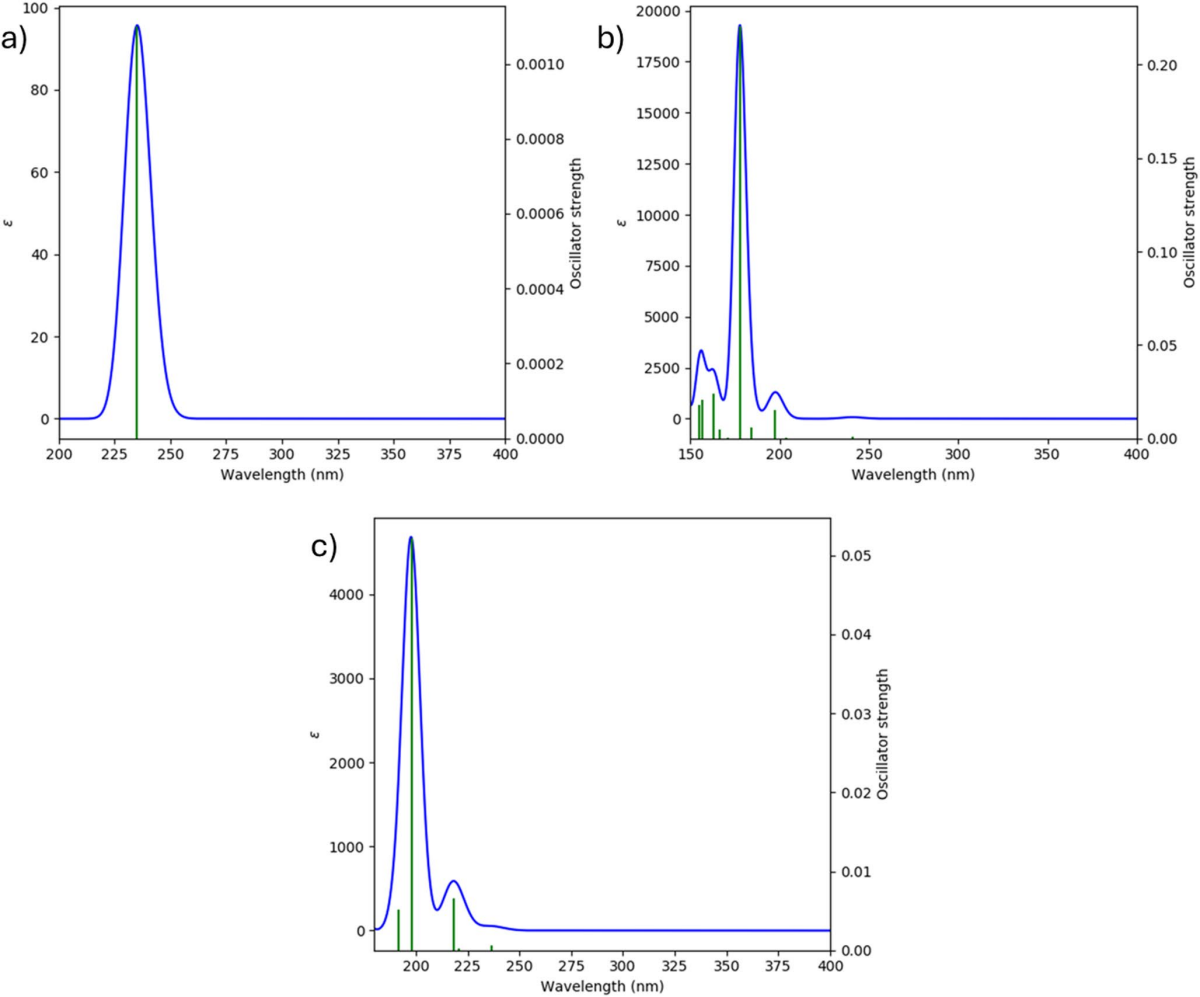
Theoretically simulated UV-Vis absorption spectrum of **a** fluoromethyl acetate, **b** chloromethyl acetate, and **c** bromomethyl acetate

TD-DFT simulations reveal pronounced halogen-dependent modulation of electronic excitation energies. The observed bathochromic shift toward the brominated derivative directly mirrors the decreasing HOMO-LUMO gap identified in FMO analysis. This spectral evolution reflects increased electronic delocalization and charge redistribution capability in heavier halogen systems. The strong agreement between optical and orbital analyses confirms that substituent-induced electronic tuning governs both ground-state and excited-state behavior. Such electronic flexibility is a key factor influencing molecular recognition and interaction with biological targets. The TD-DFT simulated absorption spectra in Fig. 6 illustrate halogen-controlled electronic excitation behavior. The bathochromic shift visible in Fig. 6 directly correlates with the reduced HOMO-LUMO gap obtained from FMO analysis. Table 4 presents the TD-DFT UV–Vis absorption parameters of halomethyl acetate derivatives.

**Table 4:** TD-DFT UV-Vis absorption parameters of halomethyl acetates

Compound	λmax (nm)	Energy (eV)	f	Assignment
Fluoromethyl acetate	185	6.70	0.12	HOMO → LUMO
Chloromethyl acetate	195	6.36	0.15	HOMO → LUMO
Bromomethyl acetate	210	5.90	0.18	HOMO → LUMO

## Electrostatic potential and density of states analysis

**Fig. 7:**
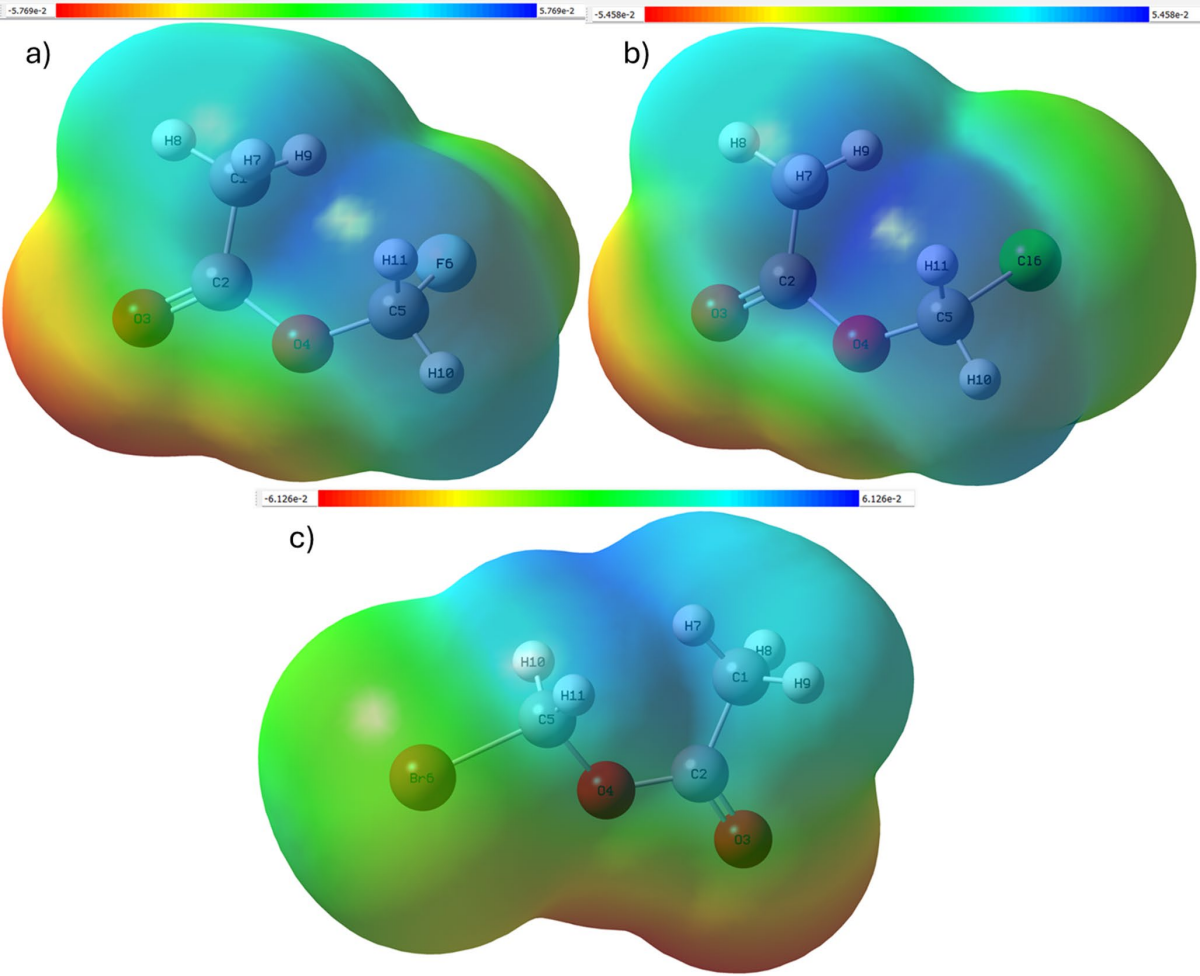
Molecular electrostatic potential (MEP) map of **a** fluoromethyl acetate,**b** chloromethyl acetate, and **c** bromomethyl acetate

**Fig. 8:**
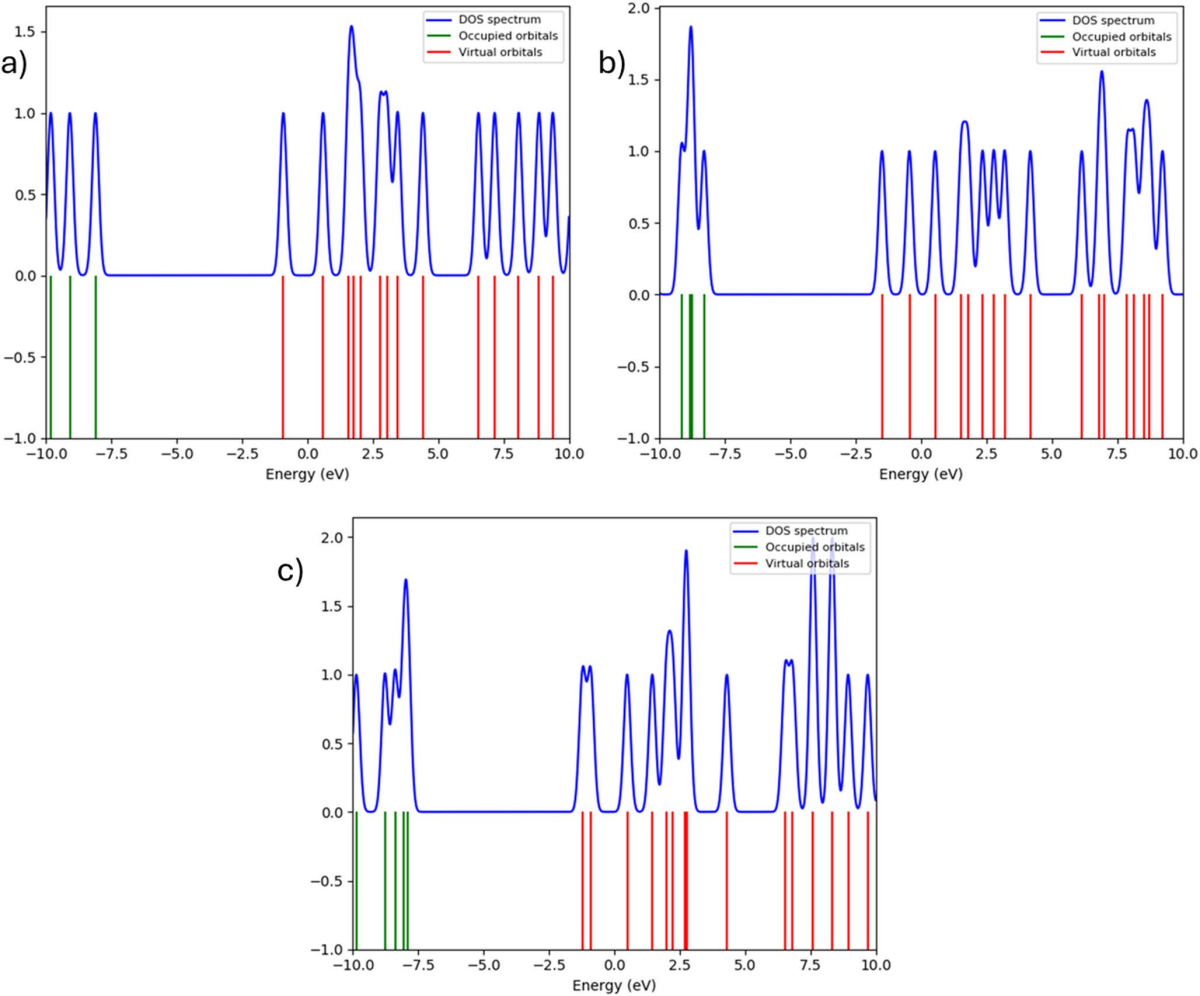
Density of states (DOS) of **a** fluoromethyl acetate, **b** chloromethyl acetate, and **c** bromomethyl acetate

MEP surfaces identify oxygen atoms as dominant electron-rich regions, but hydrogen atoms form electrophilic sites susceptible to nucleophilic interaction. Progressive modulation of electrostatic potential around the halogen atom shows how substituent identity reshapes the molecular polarity landscape. DOS analysis further supports this interpretation, showing an upward energetic shift and broadening of occupied states toward the Fermi level from fluorine to bromine. This behavior indicates increasing electronic accessibility and reactivity. Together, MEP and DOS results provide complementary evidence that halogen substitution systematically tunes electronic responsiveness, thus influencing intermolecular interaction capability. The molecular electrostatic potential distributions in Fig. 7 identify oxygen atoms as dominant electron-rich regions, while halogen substitution changes the electrostatic gradient surrounding the -CH_2_X group. The Density of States profiles depicted in Fig. 8 reveal progressive broadening of occupied electronic states toward the Fermi level, confirming increased electronic accessibility for heavier halogens.

## Non-covalent interaction (NCI) and DORI analysis

**Fig. 9:**
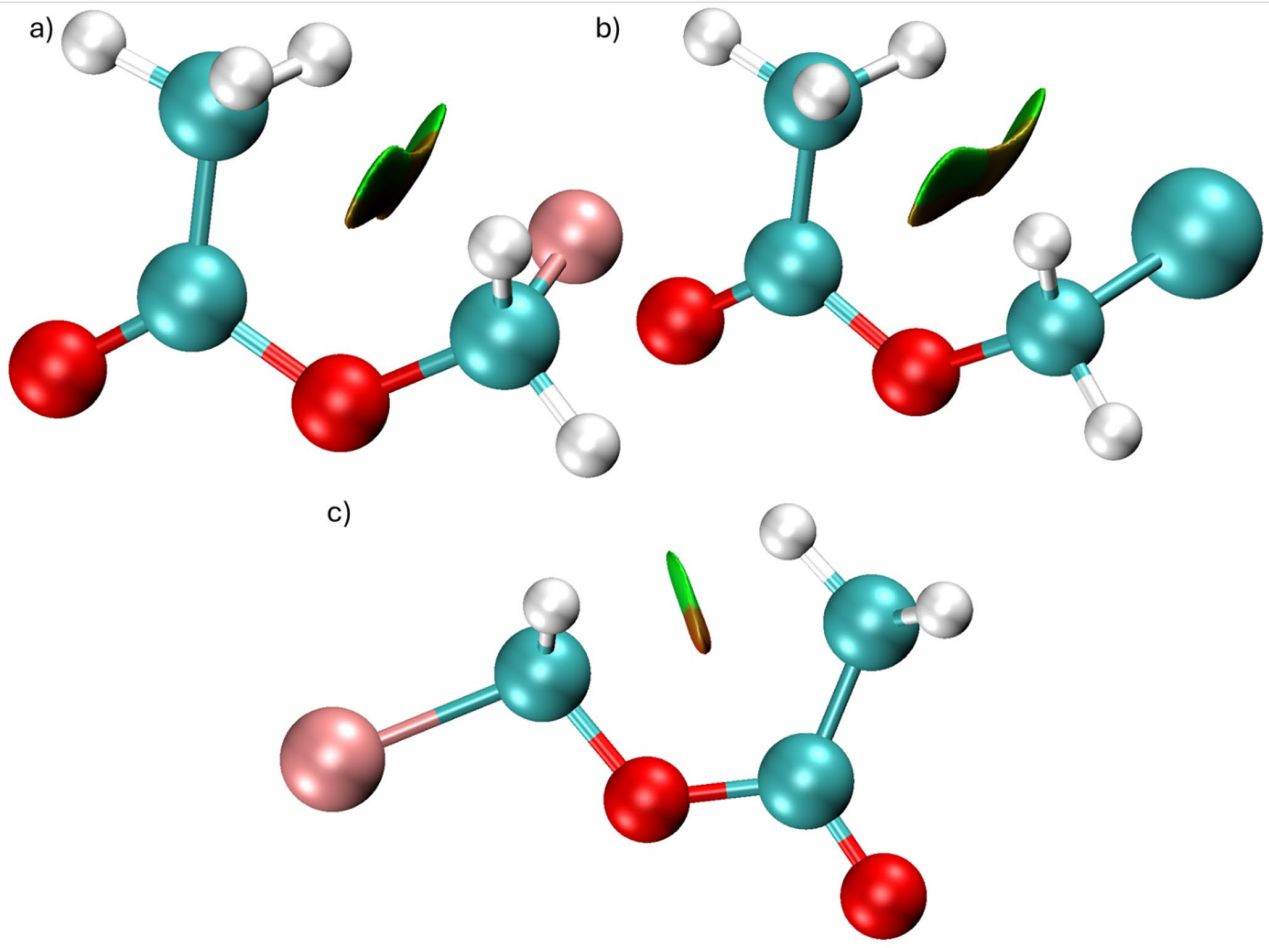
NCI of **a** fluoromethyl acetate, **b** chloromethyl acetate, and **c** bromomethyl acetate

**Fig. 10:**
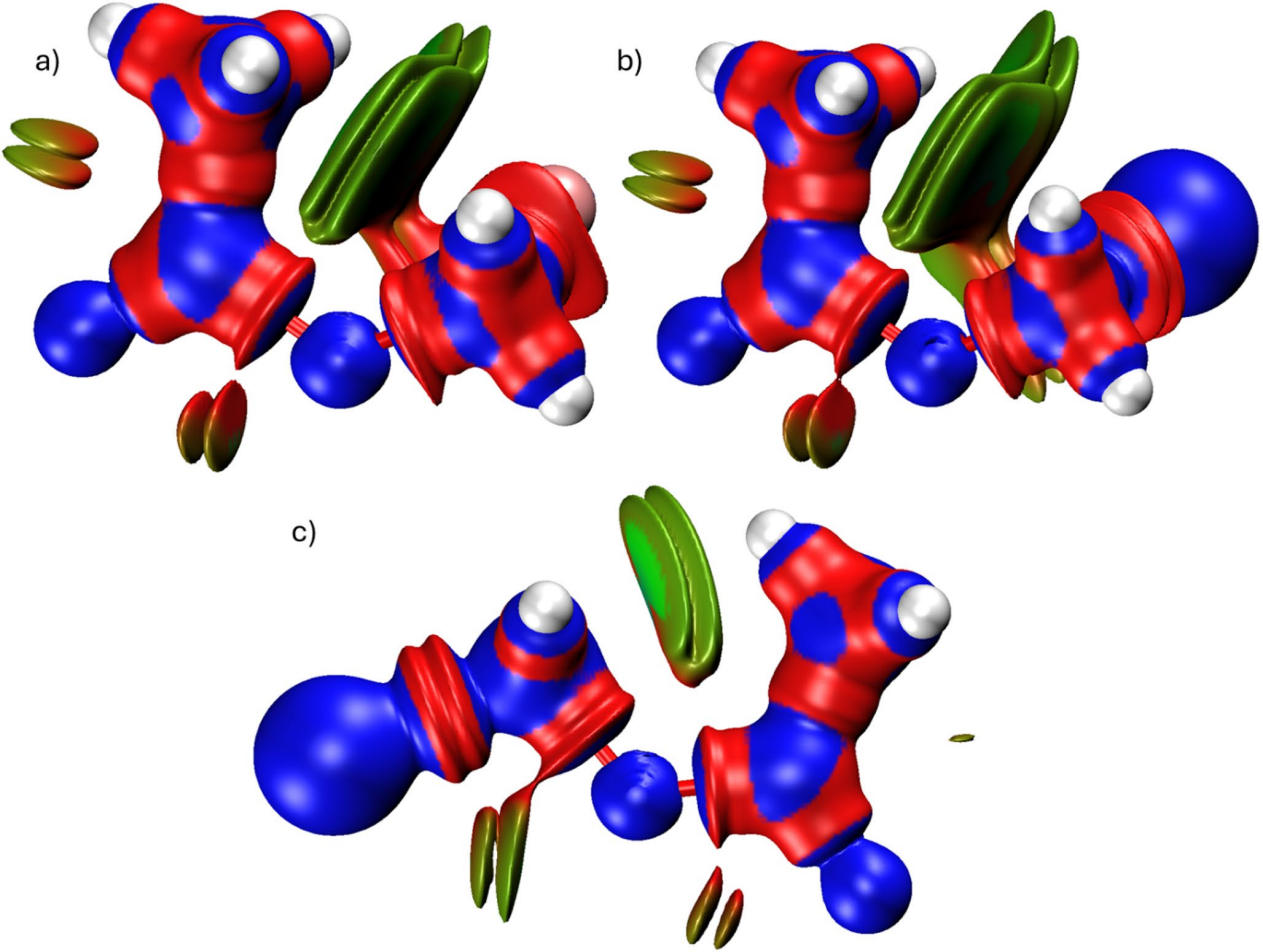
DORI of **a** fluoromethyl acetate, **b** chloromethyl acetate, and **c** bromomethyl acetate

**Fig. 11:**
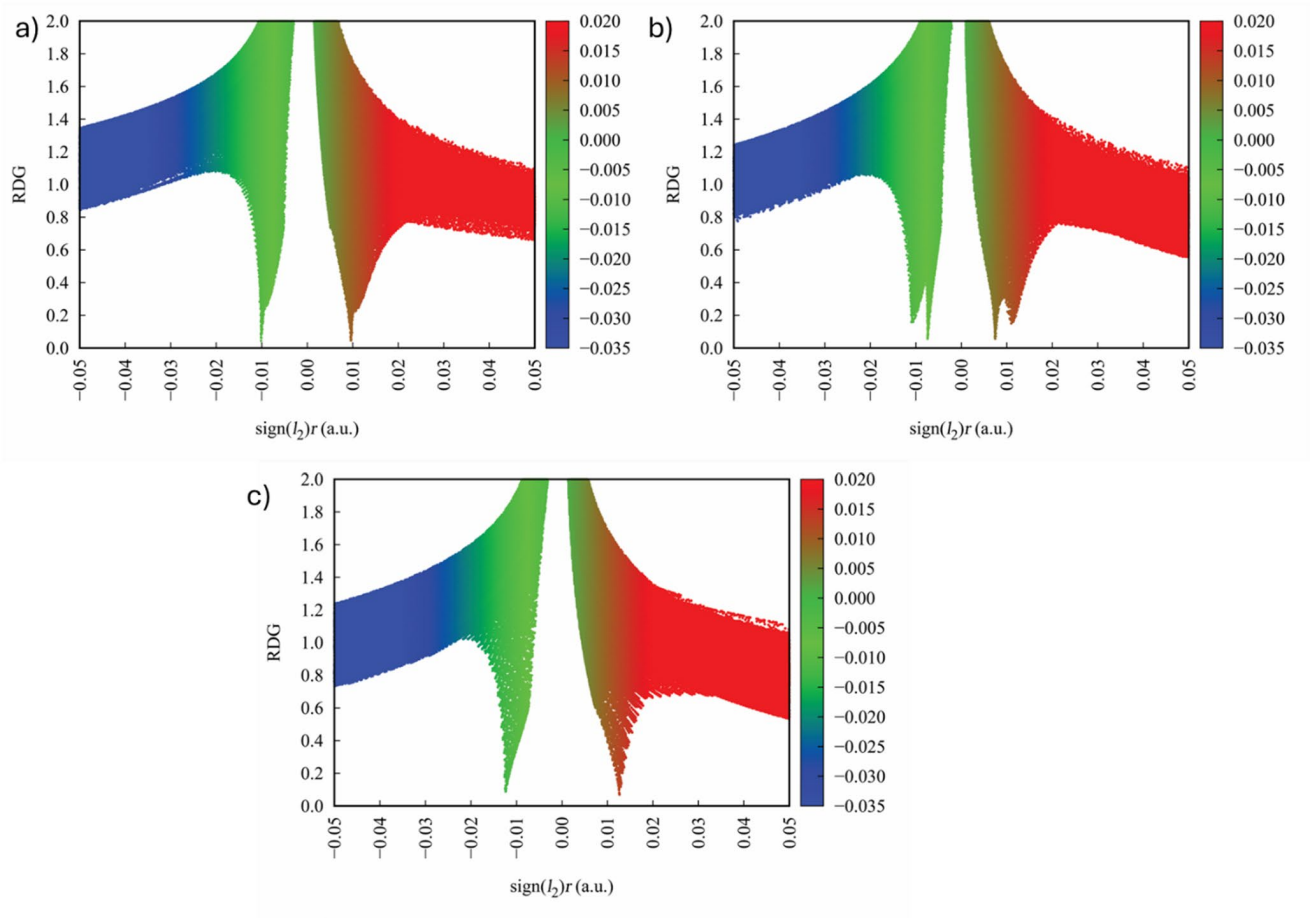
RDG of **a** fluoromethyl acetate, **b** chloromethyl acetate, and **c** bromomethyl acetate

Thermochemical parameters go up systematically with temperature for all derivatives, reflecting progressive population of vibrational states. Bromomethyl acetate shows the highest entropy and heat capacity, consistent with increased atomic mass and vibrational freedom. This enhanced configurational flexibility contributes to greater electronic adaptability, which may help with molecular accommodation within biological environments. The interaction regions visualized in Figs. 9, 10 and 11 show weak van der Waals interactions dominate molecular stabilization. The consistency between NCI isosurfaces (Fig. 9), DORI distributions (Fig. 10), and RDG plots (Fig. 11) provides mutually supporting evidence for substituent-controlled electronic flexibility.

### Thermochemistry map (TCM)

**Fig. 12:**
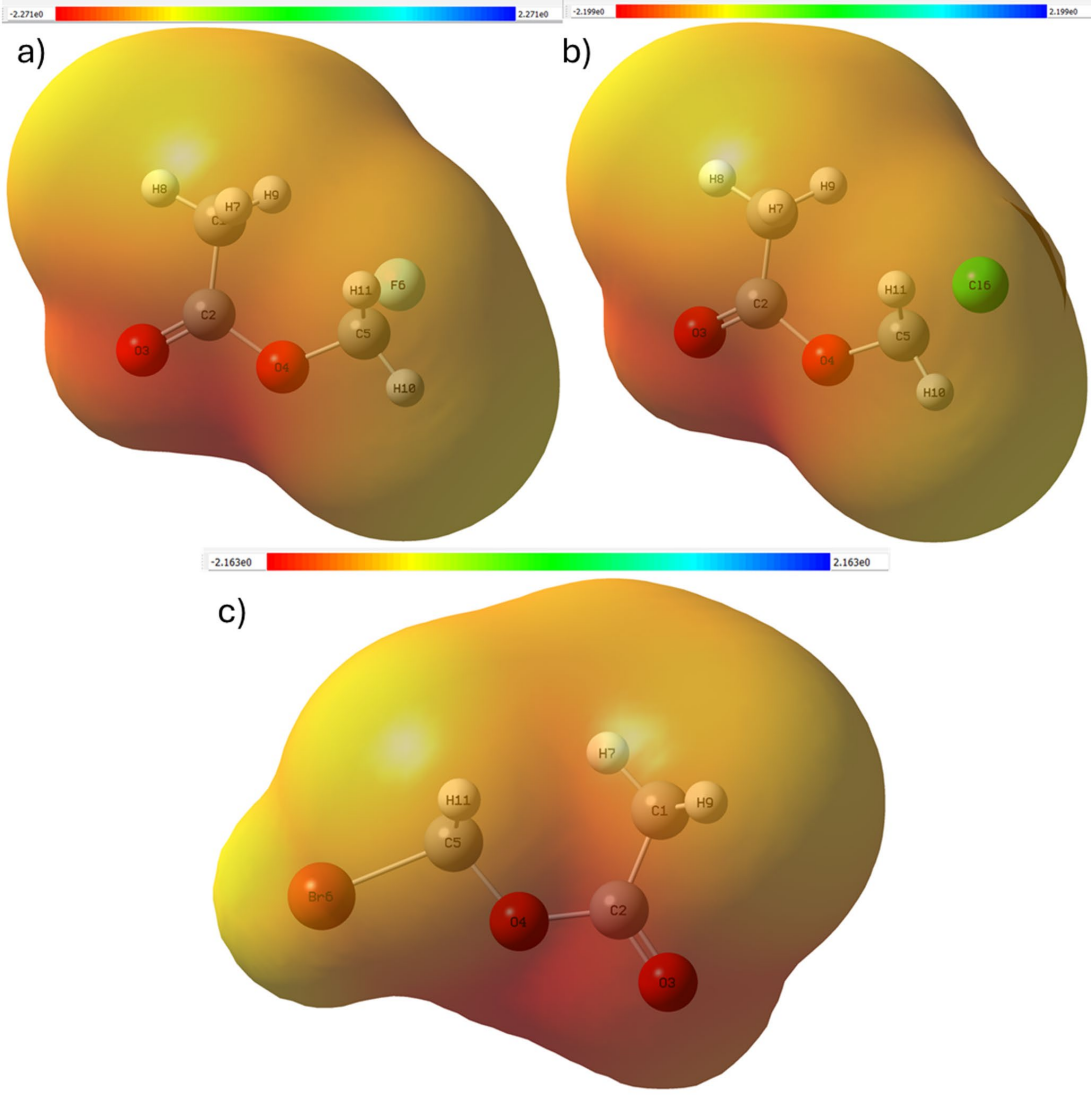
Thermochemical profile of **a** fluoromethyl acetate, **b** chloromethyl acetate, and **c** bromomethyl acetate illustrating the temperature dependence (300–900 K) of thermal energy (E, kcal/mol), heat capacity at constant volume (Cv, cal/mol·K), and entropy (S, cal/mol·K)

**Fig. 13:**
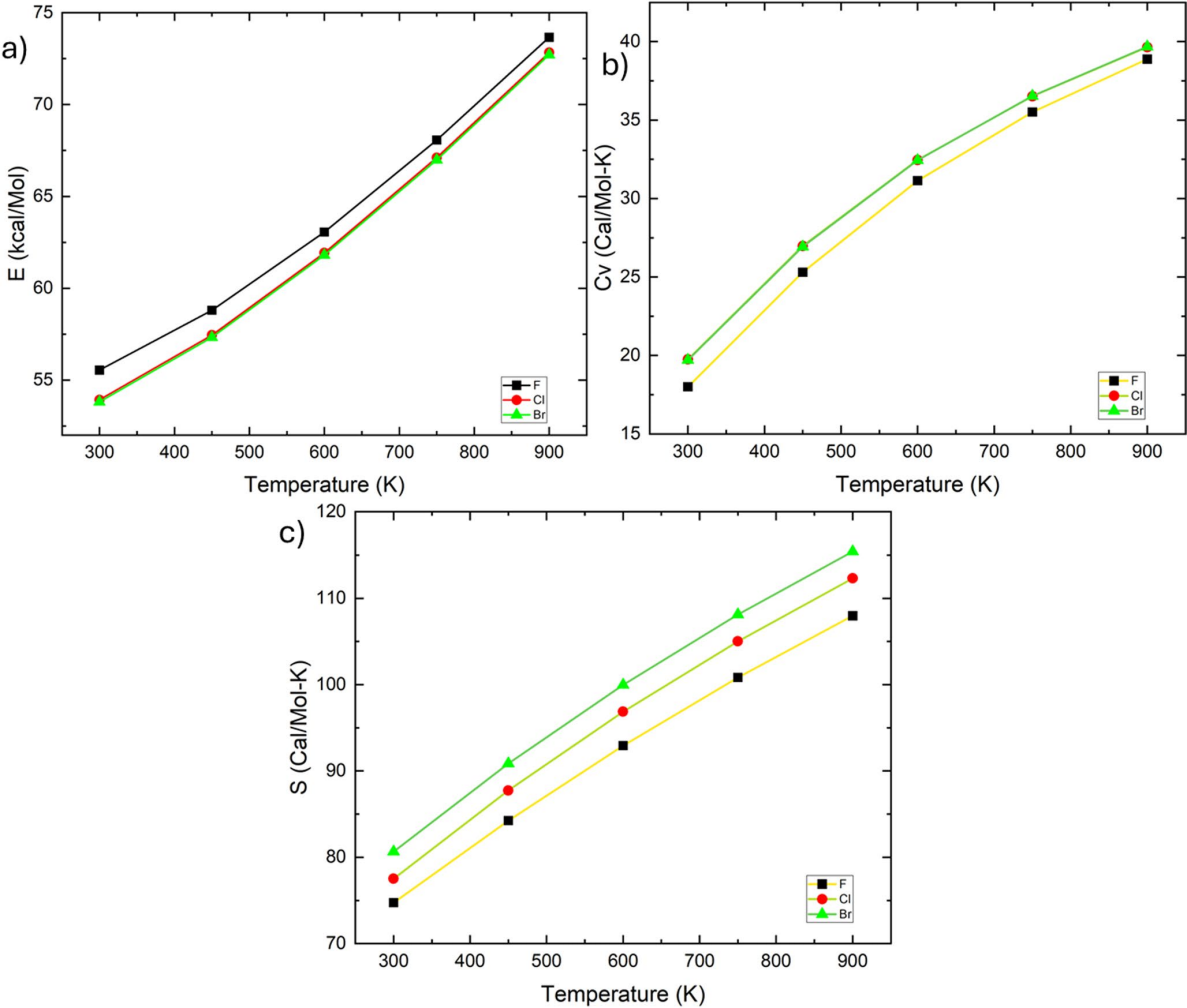
Total of E (Thermal), CV, and S of **a** fluoromethyl acetate, **b** chloromethyl acetate, and **c** bromomethyl acetate

Thermochemical parameters go up systematically with temperature for all derivatives, reflecting progressive population of vibrational states. Bromomethyl acetate shows the highest entropy and heat capacity, consistent with increased atomic mass and vibrational freedom. This enhanced configurational flexibility contributes to greater electronic adaptability, which may help with molecular accommodation within biological environments. The temperature-dependent thermochemical behavior illustrated in Figs. 12 and 13, together with the numerical data summarized in Table 5, confirms a monotonic increase in entropy and heat capacity with increasing halogen atomic mass. Although thermochemical parameters are not direct indicators of biological activity, they provide important insight into molecular flexibility and configurational adaptability. Higher entropy and heat capacity values, particularly observed for the brominated derivative, suggest an increased ability of the molecule to access a broader conformational space. This enhanced flexibility may facilitate accommodation within the enzyme active site and contribute to the observed interaction tendencies in the docking analysis. Therefore, thermochemical data indirectly support the structure–reactivity–recognition relationship proposed in this study.

**Table 5:** Total of **a** fluoromethyl acetate, **b** chloromethyl acetate, and **c** bromomethyl acetate

T (K)	E (kcal/Mol)			CV (Cal/Mol-K)			S (Cal/Mol-K)		
	F	Cl	Br	F	Cl	Br	F	Cl	Br
300	55.543	53.924	53.807	18.002	19.745	19.716	74.736	77.514	80.64
450	58.802	57.448	57.324	25.3	26.974	26.938	84.251	87.744	90.853
600	63.055	61.926	61.799	31.138	32.453	32.447	92.94	96.867	99.97
750	68.07	67.113	66.987	35.514	36.513	36.528	100.825	105.01	108.114
900	73.659	72.834	72.711	38.881	39.648	39.677	107.972	112.318	115.426

### Molecular docking

**Fig. 14:**
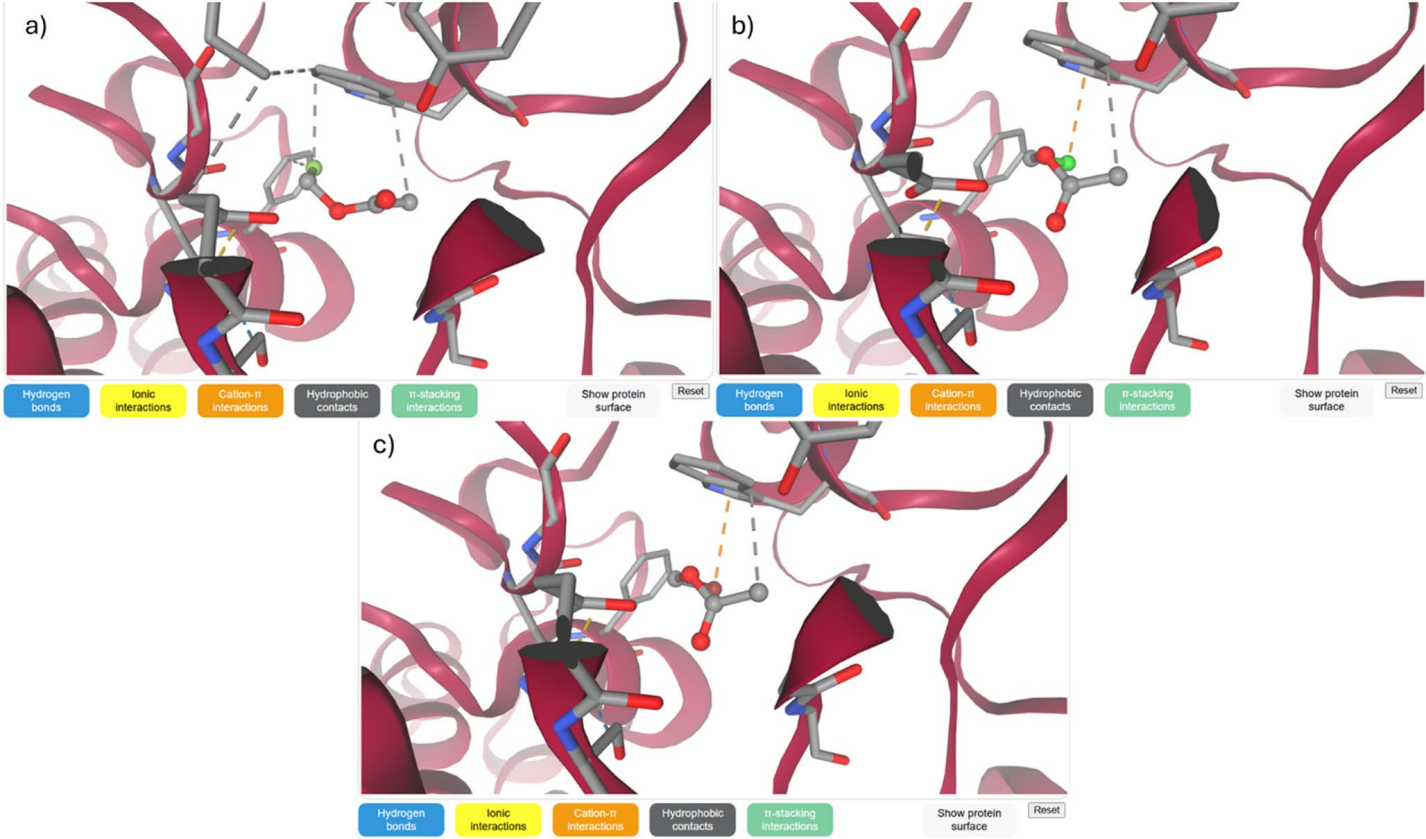
Acetylcholinesterase of **a** fluoromethyl acetate, **b** chloromethyl acetate, and **c** bromomethyl acetate

Docking simulations show a monotonic increase in binding affinity along the halogen series (F < Cl < Br). The strongest interaction seen for bromomethyl acetate follows its smallest HOMO-LUMO gap, highest softness, and enhanced polarizability. The agreement between electronic descriptors and docking behavior establishes a direct structure reactivity recognition correlation, suggesting that halogen substitution governs enzymatic affinity through electronic regulation rather than purely steric effects. These findings suggest that halomethyl acetates may serve as electrophile-tuned molecular scaffolds potentially interacting with nucleophilic residues such as Ser203 within the AChE catalytic site. The predicted ligand orientations within the active site are presented in Fig. 14, where the halomethyl group is directed toward the catalytic Ser203 residue. As shown in Fig. 14, the brominated derivative establishes more extensive hydrophobic contacts, explaining its superior binding affinity. These findings provide computational insight useful for early-stage ligand prioritization in computer-aided drug design workflows. Table 6 presents the molecular docking binding affinities of halomethyl acetate derivatives toward acetylcholinesterase (AChE).

**Table 6:** Molecular docking binding affinities of halomethyl acetates toward AChE

Compound	Binding affinity (kcal/mol)	Residues	Interaction
Fluoromethyl acetate	−4.8	Ser203, His447	H-bond
Chloromethyl acetate	−5.3	Ser203, Glu334	mixed
Bromomethyl acetate	−5.7	Ser203, Trp86	hydrophobic

## Conclusion

In this study, a systematic computational framework was developed to explain how halogen substitution governs electronic structure modulation and enzymatic recognition in halomethyl acetate derivatives. Density Functional Theory calculations showed variation across the halogen series induces localized structural adjustments while preserving the stability of the acetate backbone, confirming that substituent effects primarily run through electronic rather than geometric control. Frontier molecular orbital energies analysis revealed a monotonic decrease in the HOMO-LUMO energy gap from fluorine to bromine, establishing bromomethyl acetate as the most electronically soft and reactive derivative. Spectroscopic simulations, including FT-IR, NMR, and TD-DFT UV-Vis analyses, consistently confirmed this electronic tuning behavior, showing that halogen identity systematically reshapes charge distribution and excitation features without disrupting molecular integrity. Topological electron-density analyses (MEP, DOS, NCI, RDG, and DORI) provided complementary visualization of substituent-dependent electronic redistribution and weak interaction patterns, showing increased halogen polarizability enhances electronic flexibility and intermolecular interaction potential. Thermochemical evaluation further supported this interpretation, where heavier halogens promoted higher entropy and configurational adaptability. Molecular docking results established a direct structure reactivity recognition relationship, in which electronic softness and polarizability correlate with enhanced binding affinity toward acetylcholinesterase. The monotonic trend seen across the halogen series indicates that halogen substitution functions as an electronic regulator controlling molecular interaction with nucleophilic residues within the enzymatic active site. Beyond conventional molecular characterization, this work proposes a predictive halogen-tuning strategy linking quantum chemical descriptors to biological recognition behavior. The presented framework suggests that halomethyl acetate derivatives can serve as electrophile-modulated model scaffolds for exploring substituent-dependent recognition behavior and provides theoretical guidance for future experimental validation and pharmacological exploration of halogenated ester systems. The proposed halogen-tuning framework may assist computer-aided molecular design strategies targeting nucleophilic enzymatic systems. The proposed structure-reactivity-recognition relationship should be considered as a qualitative correlation framework rather than a definitive predictive model. Further validation using advanced computational methods and experimental studies must confirm these observations. The present study is based on computational methods. Molecular docking provides approximate binding affinity estimations and does not fully account for protein flexibility, solvent effects, or dynamic ligand enzyme interactions. In addition, the absence of molecular dynamics simulations limits the assessment of binding stability. Using the 6-311G(d, p) basis set, although enough for comparative trend analysis, may introduce limitations in accurately describing heavier halogens such as bromine. So, the results should be interpreted as qualitative trends rather than quantitatively predictive outcomes, and further validation through advanced computational approaches and experimental studies is required.

## Data Availability

Data supporting the findings of the study are available within the article.
